# Tranexamic acid‐induced fixed drug eruption confirmed by the drug lymphocyte transformation test

**DOI:** 10.1002/ccr3.2442

**Published:** 2019-09-26

**Authors:** Kenji Kawaguchi, Shotaro Kinoshita, Motonao Ishikawa, Hiroshi Sakura

**Affiliations:** ^1^ Postgraduate Clinical Training Center Tokyo Women's medical University Medical Center East Tokyo Japan; ^2^ Department of Medicine Tokyo Women's medical University Medical Center East Tokyo Japan

**Keywords:** drug‐induced aseptic meningitis, drug lymphocyte transformation test, fixed drug eruption, loxoprofen, tranexamic acid

## Abstract

We present the first case of multiple fixed drug eruption caused by tranexamic acid, which was confirmed by the LTT. This case was difficult to diagnose because the drug‐induced aseptic meningitis by loxoprofen was occurred simultaneously.

## Graphical abstract

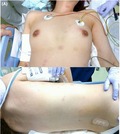

## 1 CASE REPORT

Tranexamic acid is a synthetic lysine analog that inhibits the proteolytic action of plasmin on fibrin clots and platelet receptors, thereby inhibiting fibrinolysis. It is widely used to treat various diseases, such as leukemia, hypermenorrhea, and hemophilia, and for prevention of blood loss during surgery or acute trauma. Besides its hemostatic effect, tranexamic acid is also applied in mucocutaneous diseases such as tonsillitis, angioedema, stomatitis, and urticaria.^1^ Therefore, it is sometimes prescribed with cold medicine including pharyngitis in Japan. Although some mild gastrointestinal symptoms can occur, drug eruption induced by tranexamic acid is extremely rare. To our knowledge, only nine cases of fixed drug eruption induced by tranexamic acid have been reported in Japan. These cases were positive as detected by patch and/or challenge tests only.^2^ Here, we present the first case of multiple fixed drug eruption caused by tranexamic acid, which was confirmed by the drug lymphocyte transformation test (LTT).

**Figure 1:**
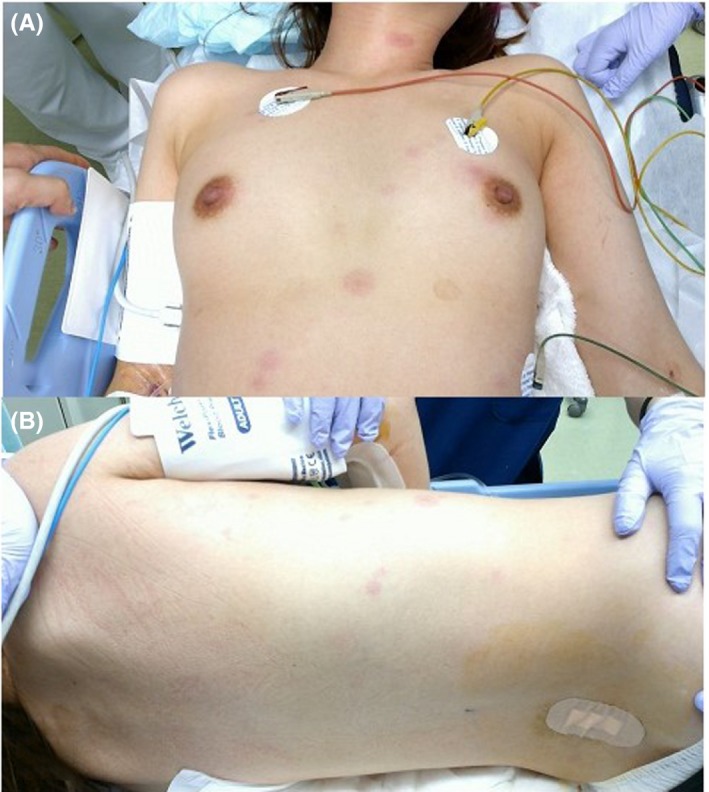
Clinical pictures of the multiple, well‐circumscribed, round, pigmented patches with surrounding erythema on the precordia, neck (A), and back (B)

A 42‐year‐old Japanese woman was admitted to the intensive care unit of our hospital with fever, moderate disorientation, and multiple, well‐circumscribed, round, pigmented patches with surrounding erythema on her precordia, neck, and back, which had appeared 2 days prior to presentation (Figure 1) after amoxicillin, tranexamic acid, and loxoprofen were prescribed for bacterial pharyngitis due to cold. Cerebrospinal fluid examination indicated a diagnosis of aseptic meningitis. Skin lesions, fever, and disorientation immediately regressed upon stopping these three drugs and with administration of 60 mg of prednisolone for 4 days. As a past history, she stated that similar eruptions had appeared 2 years ago, when she was treated with tranexamic acid for tonsillitis. Ten months later, she again developed aseptic meningitis the day after she took loxoprofen for a headache; her symptoms disappeared immediately after cessation of loxoprofen. Cutaneous symptoms were not observed throughout this period. Since the result of LTT for tranexamic acid was positive, the skin rash was diagnosed as fixed drug eruption induced by tranexamic acid. With respect to meningitis, drug‐induced aseptic meningitis by loxoprofen was diagnosed, based on immune abnormality findings, such as positive results of anti‐nuclear antibody, anti‐centromere antibody, and anti‐RNA polymerase tests, despite the lack of Raynaud's phenomenon and joint symptoms.

LTT is a minimally invasive procedure and is the first‐line diagnostic procedure when drug eruption is suspected. In this case, although LTT was performed 5 days after the discontinuation of prednisolone, it would have been better to perform LTT at least 4 weeks after treatment with prednisolone,^3^ when the positivity of LTT would have been higher. Drug‐induced aseptic meningitis may be attributable to type III and type IV hypersensitivity, which enhances lymphocyte and neutrophil activities.^4^ The patient was reluctant to undergo a biopsy and patch test, and the lack of these results could be a limitation of this case report, as LTT is not a completely validated technique.

Altogether, we should be aware that tranexamic acid, although generally a safe and frequently used drug, is associated with potential life‐threatening drug eruptions. Furthermore, detailed history taking of drug use and symptoms of collagen disease is useful for diagnosis in young women.

## CONFLICT OF INTEREST

No conflict of interest.

## AUTHOR CONTRIBUTIONS

KK: conceived and designed the case report and wrote the manuscript. SK and MI: contributed to patient management. HS: conceived and designed the case report and edited manuscript. All authors have read and approved the final draft of the manuscript.
